# Hypothesis: Etiologic and Molecular Mechanistic Leads for Sporadic Neurodegenerative Diseases Based on Experience With Western Pacific ALS/PDC

**DOI:** 10.3389/fneur.2019.00754

**Published:** 2019-07-31

**Authors:** Peter S. Spencer

**Affiliations:** Department of Neurology, School of Medicine and Oregon Institute of Occupational Health Sciences, Oregon Health & Science University, Portland, OR, United States

**Keywords:** amyotrophic lateral sclerosis, atypical parkinsonism, progressive supranuclear palsy, Alzheimer disease, DNA damage, cycad methylazoxymethanol and L-BMAA, nitrosamines, hydrazines

## Abstract

Seventy years of research on Western Pacific amyotrophic lateral sclerosis and Parkinsonism-dementia Complex (ALS/PDC) have provided invaluable data on the etiology, molecular pathogenesis and latency of this disappearing, largely environmental neurodegenerative disease. ALS/PDC is linked to genotoxic chemicals (notably methylazoxymethanol, MAM) derived from seed of the cycad plant (*Cycas* spp.) that were used as a traditional food and/or medicine in all three disease-affected Western Pacific populations. MAM, nitrosamines and hydrazines generate methyl free radicals that damage DNA (in the form of *O*^6^-methylguanine lesions) that can induce mutations in cycling cells and degenerative changes in post-mitotic cells, notably neurons. This paper explores exposures to naturally occurring and manmade sources of nitrosamines and hydrazines in association with sporadic forms of ALS (with or without frontotemporal degeneration), progressive supranuclear palsy, and Alzheimer disease. Research approaches are suggested to examine whether these associations might have etiological significance.

**Lay Summary:**

Unknown environmental exposures are thought to be risk factors for non-inherited forms of certain progressive brain diseases, such as sporadic forms of amyotrophic lateral sclerosis (sALS), progressive supranuclear palsy (sPSP), and Alzheimer's disease (sAD). Related progressive and fatal brain disorders coalesce in a single neurodegenerative disease of largely environmental origin (ALS-Parkinsonism-dementia Complex) that has affected three genetically distinct populations residing in islands of the Western Pacific region. Prolonged study of this prototypical neurodegenerative disease has provided invaluable information on the probable environmental cause (specific chemical genotoxins) and molecular mechanisms (unrepaired nerve cell DNA-damage) by which brain degeneration begins, evolves and, years or decades later, clinical signs appear, and progress. This information is used as a foundation to explore whether chemically related genotoxins (nitrosamines, hydrazines) are possible risk factors for sALS, sPSP, and sAD. Methods to test this hypothesis in the field and laboratory are proposed.

## Introduction

There is wide acceptance that environmental exposures contribute to an unknown extent to the genesis of sporadic neurodegenerative disorders. such as amyotrophic lateral sclerosis (sALS), progressive supranuclear palsy (sPSP), and Alzheimer disease (sAD). The nature of these environmental factors, when critical exposures occur, and the molecular mechanisms that propel progressive neuronal demise, are largely unknown. The one exception is Western Pacific ALS where the location of exposure is known, the nature of the culpable chemicals and their molecular mechanisms increasingly understood, and the latency (years to decades) to onset of clinical disease established. It seems logical therefore to draw on this knowledge to develop testable hypotheses about exposures, mechanisms, and course of related neurodegenerative disorders, including sporadic forms of ALS, PSP, and AD.

## Western Pacific ALS/PDC

### Characterization

Three genetically distinct populations in the Western Pacific region have experienced a very high incidence of ALS: Chamorro and other residents of Guam and Rota in the Mariana Islands; Japanese residents in two regions of the Kii Peninsula of Honshu Island, Japan, and Auyu and Jaqai linguistic groups on the island of New Guinea in Papua, Indonesia. These geographic isolates of ALS are associated with a high incidence of atypical parkinsonism (P) and dementia (D), such that they are described as the Western Pacific ALS/P-D Complex (ALS/PDC). Over the past many decades, disease incidence has declined in all three affected populations: for example, whereas in the 1950s, Guam ALS was often familial and 50–100 times more prevalent than anywhere else in the world (1), the disease has now reportedly disappeared from the island (Steele, personal communication). This occurred in concert with the post-World War II acculturation of the Guamanian population to North American practices (2).

Whereas, Western Pacific ALS/PDC was once thought to be genetic and later attributed to gene-environmental interactions (3, 4), it is now clear the etiology of this disease is heavily environmental in origin (2). The clinical phenotypes of ALS/PDC resemble a dose-response regimen in which the largest exposures precipitate motor neuron degeneration at a young age (teenage onward), parkinsonism-dementia with some amyotrophy impacts older subjects, AD-like dementia affects the oldest and putatively least-exposed clinical phenotype, while the pervasive neurofibrillary pathology in clinically normal Guamanians probably reflects the lowest level of exposure to the culpable environmental agent(s) (2). That ALS/PDC is a single disease entity is generally accepted given the presence in some subjects of overlapping clinical and neuropathological phenotypes.

### Exposure Timing, Type, and Latency

Various studies of population movement from and to areas of high-incidence ALS/PDC have provided critical information on the relationship between the duration of exposure to the disease-associated environment and the latency between the time of exposure termination and the appearance of clinical disease. Study of migrants from Guam to North America showed that risk for neurodegenerative disease could be acquired on-island before the age of 18 and appear clinically up to 34 years after arrival on the U.S. mainland (5). Similarly, Filipino migrants sometimes developed clinical ALS or parkinsonism-dementia years or decades after moving to and arrival in Guam (6). One 3-year-old Japanese subject who migrated from the southern high-risk ALS/PDC area in the Kii Peninsula developed pyramidal signs, parkinsonian symptoms, and mildly impaired cognitive function 73 years later (7), and a member of the U.S. Air Force stationed on Guam for only 4 months was diagnosed with ALS 10–20 years later (2). Taken together, these data suggest that exposure to an environment with high-incidence ALS/PDC is a risk factor for motorsystem disease that appears clinically years or decades after exposure.

While the foregoing suggests the critical environmental exposure can occur in infancy, childhood or adulthood, there is also evidence of developmental perturbation of the brains of some subjects with Guam and Kii-Japan ALS/PDC. A proportion of Guam and Japanese subjects that died with ALS/PDC in late life showed neuropathological evidence of ectopic and multinucleated Purkinje cells (8, 9) similar to those reported in postnatal mice treated with the principal cycad-derived genotoxin (10). The potential significance of these findings for ALS/PDC was discussed in 1987 by Spencer (11) in relation to “slow toxin(s)” in food prepared from incompletely detoxified water-soaked cycad seed formerly eaten on Guam. Thus, the subclinical cerebellar abnormalities found in some Guamanian and Japanese ALS/PDC cases may represent biological markers of early-life exposure to cycad toxins or chemically related substances (see below).

### Environmental Agent

The culpable environmental factor(s) appear to be linked to traditional practices that have declined with the advance of modernity in the three Western Pacific populations formerly impacted by high-incidence ALS/PDC. The only significant environmental exposure common to all three geographic isolates of the neurodegenerative disease is to the neurotoxic seed of the cycad plant (*Cycas* spp.) (2), which in various forms was used without detoxication as a traditional medicine. In addition, on Guam and Rota, cycad seed was traditionally an important source of food, especially during periods of shortage. As observed in 1954 by ethnobotanist Marjorie Whiting, preparation (detoxication) is laborious since it involves prolonged soaking of the cycad seed gametophyte in water that must be frequently changed. While a common food component at village fiestas during which all-comers (including state-siders, such as U.S. military) were welcome, “only small amounts are given to children because many became ill when they first eat a dish made with cycad starch… Some people can never eat the Federico (cycad): they get a headache even though they like it… When I inquired about the cause of ALS (or, in the vernacular “*leetiko*”), several persons suggested the cycad.” People like to eat it and although they know of its toxicity [and] go to considerable lengths to obtain and process it (12). At least one Chamorro adult with ALS/PDC reported exposure to a “bad batch” of cycad on a single occasion (*The Poison that Waits?* BBC, London. 1968. https://vimeo.com/1621281 at 40 minute mark).

In Kii-Japan, cycad seed was prescribed by folk medical practitioners (*kitoshi*) for various ailments (e.g., gonorrhea, dysmenorrhea, neuralgia, abdominal discomfort) and also used to prepare a tonic in a home-based remedy intended to strengthen the body (2). With the post-World War II introduction of allopathic medicine (Ki-Japan, Guam) and American food culture (Guam), the traditional practices declined and gradually disappeared in concert with the risk for ALS/PDC. Similarly, with the Indonesian colonization of West New Guinea, the prevalence of ALS/PDC among the Auyu and Jaqai also declined.

### Cycad Chemicals

The major neurotoxic chemical in the seed of *Cycas* spp. is cycasin, the glycone of the genotoxic and neurotoxic agent methylazoxymethanol (MAM). Also present in smaller concentrations is the neurotoxic amino acid beta-*N*-methylamino-L-alanine (L-BMAA) (Table 1), a compound that has received much research attention since my group showed in 1987 that large daily oral doses induced a motorsystem disorder in adult primates [reviewed in (13)]. Cycasin appears to be responsible for the locomotor disorder in animals grazing on cycad leaves, and there is strong experimental evidence that MAM is a potent developmental neurotoxin. The concentration in Chamorro-style cycad flour of residual cycasin, but not of L-BMAA, showed a strong statistical relationship with the historical incidence of ALS and P-D among Guam males and females (14). Whether cycasin-MAM alone or with L-BMAA contributes to the etiology of ALS/PDC is unclear but the fact that both substances can act as alkylating agents that induce DNA damage (15, 16) is of singular importance to the present hypothesis, as illustrated by MAM.

**Table 1:** PubChem links for chemical substances in order of their appearance in the text.

**Chemical**	**PubChem URL**
Cycasin (MAM-glucoside)	https://pubchem.ncbi.nlm.nih.gov/compound/5459896
Methylazoxymethanol (MAM)	https://pubchem.ncbi.nlm.nih.gov/compound/6433205
Beta-*N*-Methylamino-L-alanine (BMAA)	https://pubchem.ncbi.nlm.nih.gov/compound/105089
*O*^6^-Methylguanine (*O*^6^-mG)	https://pubchem.ncbi.nlm.nih.gov/compound/65275
Streptozotocin	https://pubchem.ncbi.nlm.nih.gov/compound/29327
*N*-Nitrosodiethylamine	https://pubchem.ncbi.nlm.nih.gov/compound/5921
*N*-Nitrosoatrazine	https://pubchem.ncbi.nlm.nih.gov/compound/108077
Gyromitrin	https://pubchem.ncbi.nlm.nih.gov/compound/9548611
Monomethylhydrazine (MMH)	https://pubchem.ncbi.nlm.nih.gov/compound/6061
*N*-Methyl-*N*-formylhydrazine (MFH)	https://pubchem.ncbi.nlm.nih.gov/compound/12962
*N*-Nitrosodimethylamine	https://pubchem.ncbi.nlm.nih.gov/compound/6124
Formaldehyde	https://pubchem.ncbi.nlm.nih.gov/compound/712
Nitromethane	https://pubchem.ncbi.nlm.nih.gov/compound/6375
Annonacin	https://pubchem.ncbi.nlm.nih.gov/compound/354398
Nitrophenylethane	https://pubchem.ncbi.nlm.nih.gov/compound/118364596
3-Nitrotyrosine	https://pubchem.ncbi.nlm.nih.gov/compound/65124
Phenylhydrazines	https://pubchem.ncbi.nlm.nih.gov/#query=Phenylhydrazines
Pyrazolones	https://pubchem.ncbi.nlm.nih.gov/#query=Pyrazolones

*PubChem is a database of chemical molecules, their activities in biological assays, and their health effects in humans and animals. The system is maintained by the National Center for Biotechnology Information (NCBI), a component of the National Library of Medicine, which is part of the U.S. National Institutes of Health (NIH)*.

### DNA Damage From Cycad Chemicals

Experimental animal studies show that single systemic doses of MAM can induce dysplasia of the developing cerebellum, features that are represented in a proportion of Guamanian and Kii-Japan subjects with ALS/PDC (8, 9). The cerebellar pathology appears to result from MAM-induced DNA damage in the form of *O*^6^-methyguanine (*O*^6^-mG) (Table 1), which is subject to repair by *O*^6^-mG methyltransferase (MGMT). MAM-induced cerebellar damage is increased in transgenic animals lacking MGMT and decreased in animals overexpressing MGMT (15). The adult human brain often has very low levels of this critically important DNA-repair enzyme such that, in MAM-treated *mgmt* ko mouse brain, DNA damage accrues and activates cell signal pathways associated with human neurodegeneration, notably AD and ALS (17). Failure of adult neurons to repair MAM-induced DNA damage is proposed as the trigger that initiates the slow process of post-mitotic neuronal demise. This is consistent with the conclusion from traditional epidemiological reasoning that cumulative DNA damage may contribute to disease onset in ALS (18, 19).

### Nitrosamines and Hydrazines

Two classes of chemicals, nitrosamines and hydrazines, have genotoxic mechanisms and clinical effects comparable with those of MAM. Like MAM, both substances are acutely hepatotoxic and potentially carcinogenic because, in mitotic cells, such as the lining of the gastrointestinal tract, overwhelming DNA damage with limited DNA repair can lead to mutation and uncontrolled cell division. In fact, azoxymethane, which is metabolized to the developmental neurotoxin MAM, is a useful experimental tool to create a model of human colon carcinogenesis (20, 21). By contrast, in the murine brain, post-mitotic neurons accrue MAM-induced DNA damage, with short and long-term effects on cell signal pathways linked to neurodegenerative disease as well as cancer (22, 23).

#### Nitrosamines

There is strong evidence for the neurotoxic potential of the nitrosamine-related compound streptozotocin (STZ: 2-deoxy-2-(3-methyl-3-nitrosoureido-D-glucopyranose) (Table 1), the cytotoxicity of which is mainly due to DNA alkylation, including O^6^-methylguanine, 7-methylguanine, 3-methyladenine and 7-methyladenine in liver, kidney, intestine, and pancreas, but little methylation of brain DNA (24). However, intracerebroventricular injection of STZ produces effects that resemble molecular, pathological, and behavioral features of AD, including increased amyloid-β protein and neurofilament expression, increased phosphorylation of tau protein, and neuronal loss in hippocampus and cerebral cortex (24–28). Rats treated with STZ show a reduction in alpha and gamma motor neurons innervating soleus and medial gastrocnemius muscles, together with the areas of corticospinal tracts serving the trunk, hindlimb and, to a lesser extent, the forelimbs (29–31). It is not clear if loss of motor neurons represents a direct effect of STZ or whether it results from a chronic diabetic state induced in the animals by the toxic effect of the nitrosamine derivative on pancreatic beta-islet cells.

The direct neurotoxic property of *N*-nitrosamines has been demonstrated in the case of the food and water contaminant *N*-nitrosodiethylamine (NDEA). Treatment of post-mitotic rat cerebellar neurons (48 h) *in vitro* produced dose-dependent increases in DNA damage and oxidative stress, similar to the effects of STZ treatment and AD neurodegeneration (32). The authors of this study pointed out that nitrosamines such as NDEA readily form in meat and fish that have been preserved or flavored with nitrites, and nitrosamines can form in the gastric acidic environment from ingested nitrites and nitrates (33–35). They suggest that the expanded use of nitrites and nitrates in foods and agricultural products over the past 30–40 years may have contributed to the growing prevalence of AD and possibly other forms of neurodegeneration (32). Exposure to nitrosamines with DNA adduct formation also occurs during inhalation of tobacco smoke, which is sometimes identified as a risk factor for both ALS (36–38) and AD (39, 40) as well as cancers (41).

Nitrosamine exposure might also be relevant to the high incidence, younger age of onset, and clustered appearance of ALS among soccer players in Italy (42–46) and elsewhere (47, 48). For Italian professional soccer players aged 45 years and younger, the ALS rate (1959–2000) was 4.7 times higher than the general population, and the average age was 43 years at onset compared with 63 years. Herbicides used on playing fields were among the factors linked to the high rates of ALS among Italian soccer players (45). Herbicides such as atrazine, which was used in Europe between 1960 and 2004, when it was banned because of persistent groundwater contamination, were employed to control weeds during both the soccer and off season (49). Atrazine can react with nitrite to form a potentially toxic nitrosamine product, *N*-nitrosoatrazine (Table 1) (50, 51). Since nitrosamines are present in rubber products (52), nitrosamines are also liberated from recycled rubber crumb used in artificial turf (53, 54). Additionally, since nitrosamines are associated with leather products (see below), contact with leather soccer balls and footwear provide other potential opportunities for exposure to these toxic substances. Professional soccer now employs synthetic leather balls.

#### Hydrazines

The second group of compounds related mechanistically to MAM are hydrazine compounds. Hydrazine is used in agricultural chemicals (pesticides), chemical blowing agents, pharmaceutical intermediates, photography chemicals, boiler water treatment for corrosion protection, textile dyes, and as fuel for rockets and spacecraft. These neurotoxic compounds are also associated with some poisonous mushrooms that are eaten and sometimes precipitate acute neurotoxic illness.

Hydrazones (such as gyromitrin: acetaldehyde *N*-methyl-*N*-formylhydrazone) that generate monomethylhydrazine (MMH) upon hydrolysis are components of certain wild fungi, notably False Morels (*Gyromitra, Helvella, Verpa* spp.) (Table 1). Consumption of *Gyromitra* spp. can trigger acute gastrointestinal (nausea, vomiting, diarrhea) and neurotoxic effects (headache, vertigo, ataxia, fever, muscle fasciculation, seizures, coma) (55). MMH forms hydrazones with pyridoxal phosphate, which reduces production of the inhibitory neurotransmitter (GABA) via decreased activity of glutamic acid decarboxylase (56). Additionally, *N*-methyl-*N*-formylhydrazine (MFH), which is produced during MMH metabolism, undergoes cytochrome P450-regulated oxidative metabolism and, via reactive nitrosamide intermediates, leads to the formation of DNA-damaging methyl free radicals (57). As with MAM and nitrosamines, the *O*^6^-methylation of guanine can cause liver and kidney lesions (58–63) and tumor formation (62). The risk of long-term adverse effects may be greater in individuals with genetic slow acetylation rates because decreased detoxication (acetylation) of MFH would result in larger amounts of MMH formed from gyromitrin (64, 65).

Since hydrazines and MAM induce the same type of DNA damage, both potentially with the involvement of formaldehyde (18, 66), it is hypothesized that single or repeated exposure to methyl free-radical-generating hydrazines might trigger long-latency neurodegeneration culminating in ALS or a related brain disease. Four sources of information are consistent with this hypothesis and thus require detailed research exploration. Two involve potential links to False Morel mushrooms and two to engine fuels, both of which are associated with hydrazines. The information available on these topics is scattered, far from complete, and is offered here with the intent of stimulating new avenues of investigation of possible risk factors for sporadic neurodegenerative disorders, the causes of which have so far defied explanation.

### ALS and Fungal Hydrazines

#### France

Mushroom consumption has been identified as a risk factor for a cluster of 12 ALS patients, one with ALS-parkinsonism, in a morel-consuming community in Savoie in the French Alps (67, 68). Between 1991 and 2003, five long-term residents (including 2 spouses) in a population of 200 subjects developed ALS. Seven other ALS cases spent part of their life in the area. Half of the 12 cases consumed local mushrooms, including morels. While True Morels (*Morchella* spp.) are highly prized as a delicacy in Europe and beyond, it is often difficult to distinguish anatomically between True and False Morels (69, 70). As described above, False Morels are a source of neurotoxic and DNA-damaging MMH. Almost half of the ALS cases had an acute intoxication with “morels” in their medical history (68).

#### Finland

The birth location of a cluster of ALS subjects in Finland corresponds to a region of False Morel consumption. A single significant cluster of 227 ALS cases was identified in southeast Finland using data on the place of birth (*circa* 1920) (71, 72). The cluster involved a population of half a million subjects residing in parts of the provinces of Kuopio, Mikkeli, and Pohjois-Karjala, as well as parts of present-day Russian Karelia. Whereas, the prevalence of multiple sclerosis was 52.5% lower, ALS rates were 225% higher among Finnish WWII evacuees from Karelia (18 per 100,000) compared with non-evacuees (8 per 100,000). As noted by the authors, these data speak against a genetic etiology for ALS and for one or more environmental factors that made the evacuees more liable to develop motor neuron disease later in their lives (73, 74).

During and after the Finnish Winter War with Russia (November 30, 1939–March 12, 1940), there was a mass migration of Karelians (*circa* 400,000 persons) to Finland and particularly to the southeast (Itä-Suomi) where there is a strong mushroom-eating culture, including MMH-generating *Gyromitra esculenta*, among Karelians. Between 1914 and 1945, one quarter of the number of acute poisonings attributed to *G. esculenta* occurred in southeastern Finland (75). Notably, at that time, dried, or once-boiled fresh specimens were considered safe to eat, in contrast to the extensive washing and double-boiling procedure later recommended by the Finnish Food Authority which, in 2019, stated that *G. esculenta* is not to be eaten by pregnant and breastfeeding women and children because of “residues of the toxin gyromitrin despite processing” (https://www.ruokavirasto.fi/en/private-persons/information-on-food/instructions-for-safe-use-of-foodstuffs/safe-use-of-foodstuffs/). Consumption of mushrooms increased during wartime because of marked food shortages. Mushroom consumption by Finnish “families of workers and functionaries” peaked between 1943 and 1944 when, in Kuopio for example, an amount of 165.5 g/week per consumer unit was 5 times greater than mean consumption in 1941 and 1945 (75).

Given the varied phenotypes of Western Pacific ALS/PDC, including an AD-like form, it is noteworthy that Finland is also reported to have the highest death rate from dementia in the world, which has been suggested to result from AD-related environmental factors, including L-BMAA generated by cyanobacteria in the Gulf of Finland and in the country's many freshwater lakes (76). A possible contribution from the unique practice of consuming wild and commercially available False Morels is another possible subject for investigation.

#### Mid-West, USA

There is a high prevalence of ALS in the mid-West (77), where cooking and eating False Morels is practiced (70, 78). The highest number of historical reports of acute MMH poisoning has occurred in Michigan (79). While environmental exposures linked to ALS have been studied in Michigan (77, 80), and in a small ALS focus near Lake Michigan WI in which familial cancer and frequent fish consumption were more often reported in cases than controls (81), the question whether sALS is linked to food use of wild mushrooms and mushroom poisoning has not been explored.

### ALS and Hydrazine Fuels

Hydrazines have been used in hypergolic fuels to power spacecraft for over 50 years (82). In the Titan II Nuclear Missile program, the toxic breakdown products of hydrazine and unsymmetrical dimethylhydrazine (UDMH) (including *N*-nitrosodimethylamine and formaldehyde) persisted for 6 weeks or longer in the missile silos after spills or leaks (Table 1) (83, 84). Ground fueling of spacecraft with hydrazine mixtures requires a crew of five using protective gear, supported by 20 specialists (84). Hydrazine fuels appear to be of past and ongoing interest to the NASA Ames Research Center in relation to the Pioneer 10 and future Mars space probes, respectively (85–89). NASA Ames lists five papers researching hydrazine sulfate and ammonium hydrazinium sulfate between 1993 and 1998 (90). In 2016, an apparent cluster of ALS cases was reported at the Ames Research Center, at Moffett Federal Airfield in California's Silicon Valley. Several of the ALS cases worked together, and seven of the 8 employees who had worked in buildings 240, 244, or 245 on the north side of the Ames campus had contracted ALS post-2000 (91, 92). Given that ~2,500 people worked at the Ames campus, the number of ALS cases was clearly in excess. One subject was diagnosed with ALS at the age of 55, 21 years after beginning work at the Ames Research Center (92). Whether there is any relationship between ALS and exposure to hydrazine or other chemicals at this site has yet to be investigated.

Hydrazine has also been used to fuel the emergency power units (EPU) of the NASA Space Shuttle, the U-2 spy plane, the International Space Station (see also Note Added in Proof), and the General Dynamics F-16 fighter jet. A 70% aqueous solution (H70) of hydrazine powers the F-16 EPU, which provides emergency electrical and hydraulic power in the event of engine failure. Pilots and ground maintenance crews can be exposed to hydrazine vapors during operations (93–96). F-16s were used by the USA between 25 January and 28 February 1991 in the Desert Storm combat phase of the Gulf War. Military personnel, notably Air Force personnel, who were deployed to the Gulf Region during the Gulf War period, experienced a greater post-war risk of ALS (and younger age of onset) than those who were not deployed to the Gulf (97). For example, one pilot who flew 44 F-16 combat missions over Iraq during Desert Storm was diagnosed with ALS at the age of 37 (98). Whether these associations are circumstantial or otherwise, they offer a new avenue to explore the etiology of early-age onset of ALS among military personnel who served in the Gulf War.

Hydrazine and nitromethane were used to fuel racing cars, funny cars and dragsters in the 1960s (99, 100). An Internet search reveals several individuals with a history of motor racing who developed ALS (101–106).

### Genotoxins and PSP and ALS

#### PSP in Guadeloupe

Typical ALS and, in older subjects, upper and lower motor neuron signs in combination with supranuclear palsy, parkinsonism, and dementia, have been described in the Guadeloupe archipelago (107). A significant number has a syndrome resembling progressive supranuclear palsy (PSP) (108, 109), which has also been reported in New Caledonia and on Guam (110–112). The disorders in Guadeloupe and New Caledonia are associated with regular food and beverage use of the fruit of Annonaceae, such as the sour sop (*Annona muricata*). Annonaceae contain the acetogenin annonacin (Table 1), a mitochondrial Complex 1 inhibitor with neurotoxic properties (113). There is also evidence that prolonged (12 months) oral ingestion of commercially available *A. muricata* juice aggravates cerebral tau phosphorylation in wild-type and tau-transgenic mice. The brains of these animals showed increased reactivity of 3-nitrotyrosine (Table 1), which suggests oral intake of sour sop juice promotes the generation of reactive nitrogen species (114). Conceivably, this might arise from the presence in Annonaceae of nitrophenylethane (Table 1) (115) which has a terminal O=N=O group that, as with nitrite, can react readily with amines and amides to form *N*-nitroso compounds (116). The possibility that MAM-like nitrosamines contribute to the etiology of environmental PSP may merit investigation see below).

#### PSP in France

Proposed as the most compelling case for an environmental etiology of PSP is the report of a cluster of classical cases in northern France (117). Cases were found in the adjacent French towns of Wattrelos and Leers, the former location throughout most of the 20th century of numerous textile-dyeing and leather-tanning plants. Area residents raised fruits and vegetables for home consumption and for sale at local markets. While the authors of this report tentatively attributed the cluster of PSP to metals present in soil—more likely to arsenic than chromium, arsenicosis and peripheral neuropathy, not PSP, usually result from chronic arsenic exposure. However, it is noteworthy that arsenic can upregulate nitrosamine metabolism and increase DNA lesions of the type associated with MAM (118).

The textile dyeing and finishing industry has utilized thousands of chemicals and is considered to have been one of the most environmentally polluting industries. A large amount of water is used to wash dyed and printed textiles and to clean printing screens and dyeing vessels. Since contaminated water has been discharged directly into water bodies, hazardous chemicals can readily enter soil (119). Among chemicals used in the dyestuff industry are phenylhydrazines that produce heterocylic coupling components such as pyrazolones (Table 1) (120). The preferred hydrazines are those containing two hydrocarbon groups bound to the same nitrogen atom, such as *N*,*N*′-dialkylhydrazines (121). Hydrazine is a reducing agent for many transition metals and some semimetals, including arsenic (122), and plants and fungi can bioconcentrate metal and non-metal substances in soil and water, including arsenic (122–126).

Leather tanneries have also used dimethylamine sulfate, a *N*-nitrosodimethylamine precursor, as a depilatory agent in the hide process (127). Thus, workers in the textile and leather industries may be exposed to nitrosamines and hydrazines. Research is therefore needed to explore the possibility that genotoxic chemicals previously used in the textile and leather industries are risk factor for sporadic forms of PSP. The textile industry in Israel (128) and the leather industry in Britain have been associated with an increased risk for ALS (129, 130), and “textile, garment, and related trade workers” were prominent among the higher incidence of ALS among female workers (131).

### DNA Damage and Repair

The foregoing shows that natural and synthetic hydrazines, nitrosamines and MAM are genotoxic chemicals that damage DNA and may be associated with neurodegenerative diseases (ALS, PSP, AD), as well as with cancer. DNA damage and repair has been critical to understanding the pathogenesis of various cancers and, in recent years, the subject has occupied increasing attention in relation to brain aging and progressive neurodegenerative disorders, including ataxia-telangiectasia, Huntington disease, Parkinson disease, AD and ALS (132) (see also Note Added in Proof).

The cellular DNA damage response (DDR) system (133, 134), which comprises at least 450 proteins, is responsible for initiating DNA repair or, if DNA damage is too severe, instructing the cell to cease growth or die. DDR proteins include Fused in Sarcoma (FUS), which is involved in DNA repair and, in mutant form, accumulates in familial ALS. FUS has marked functional similarities to TAR DNA-binding protein 43 (TDP-43), which has recently been shown also to participate in DNA repair (135). Pathological forms of TDP-43 characterize the motor neurons of almost all cases of familial ALS with frontotemporal degeneration (FTD) and approximately half of FTD cases (136, 137). TDP-43 inclusions are also present in the tau-dominated polyproteinopathy of Western Pacific ALS/PDC (138, 139), in particular in the spinal cord, limbic, and cortical regions of Guam cases (140, 141). In sum, therefore, components of the DDR response are prominent in both genetic and environmental forms of these neurodegenerative disorders. In addition to the various types of DNA damage and plasticity associated with ALS (142), evidence from Western Pacific ALS/PDC suggests that CNS *O*^6^-mG DNA damage, its specific repair enzyme MGMT, and other targets of genotoxic alkylating agents (143) merit increased research attention in this and related neurodegenerative diseases.

## Testing the Hypothesis

This hypothesis proposes that exposure to natural or manmade nitrosamines and hydrazines, acting via a specific pattern of poorly repaired neuronal DNA damage induced by methyl free radicals (144–148), may be potential risk factors for some sporadic forms of ALS, PSP, and AD. The latency of years or decades that intervenes between exposure to an environmental agent capable of inducing neurodegeneration and the appearance of clinical disease makes proof of causation exceptionally difficult. Four possible research approaches may be useful.

The first involves extensive assessment of lifetime total chemical exposure (exposome) using investigative techniques that go far beyond those typically employed in epidemiological instruments. Targeting individual cases who develop disease at a very young age has proved invaluable in past investigations of ALS/PDC because the duration of potential exposure is relatively short and living caregivers and relatives can provide information on exposures that occurred *in utero* and during infancy and early childhood of the disease-affected subject. Detective research of this type was crucial in first linking ALS in Guam and Kii-Japan to oral exposure to cycad seed (13, 149).

Secondly, a multidisciplinary research approach is needed to understand how methyl free radicals derived from alkylating agents generate *O*^6^-mG lesions that might trigger neurodegenerative disease. The low levels of the *O*^6^-mG DNA repair enzyme MGMT in human brain tissue, and its epigenetic regulation (150–152), appear to be of central importance in neuronal degeneration induced by alkylating agents such as MAM and related compounds. MGMT is a nuclear protein that transfers the alkyl group from the *O*^6^ position of guanine, thereby restoring guanosine to its undamaged state, while the alkylated “suicide” protein is subsequently ubiquitinated and then degraded by the proteasome (151, 152). However, alkylated *O*^6^-guanine lesions in rat brain genomic DNA are removed more slowly than those in liver or kidney (153–155), and MAM-induced brain DNA damage can modulate cellular pathways associated with neurodegenerative disease (17, 22, 23) and induce degeneration in non-cycling cells (156) as well as terminally dividing neurons. The amount and persistence of unrepaired DNA damage in neurons, a reflection of genotoxin dosage and DNA-repair efficiency, predictably would determine the rate of differential neuronal loss, the duration of the latency period and, potentially, the eventual clinical disease phenotype.

A third research approach concerns the possibility that metabolic differences are important in the expression of neurodegenerative disease linked to the alkylating genotoxins discussed here. For example, genetic control of the rate of metabolic acetylation (fast, intermediate, slow), a subject relevant to ALS (157), should modulate individual susceptibility to hydrazines. Similarly, aldehyde dehydrogenase (*ALDH2*), which is linked to AD risk (158), metabolizes formaldehyde, a common and potentially genotoxic metabolite of MAM and L-BMAA (17, 19).

Finally, the availability of identified single-cell sequencing provides an opportunity to determine whether DNA damage accrues in neurons vs. glial cells in sporadic neurodegenerative diseases (e.g., sALS, sPSP, sAD), and the relationship between DNA damage, gene expression, and abnormal protein deposition (159). This approach could be applied to Western Pacific ALS/PDC as well as related sporadic neurodegenerative disorders.

## Note Added in Proof

A mixture of UDMH (1,1-dimethylhydrazine) and nitrogen tetroxide is the propellant used for thruster firings on the International Space Station (ISS). The Spacecraft Maximum Allowable Concentration (SMAC) for human exposure (based on alterations of “red blood cell mass”) is set at 4 ppm (for 1 h), 0.3 ppm (24 h), 0.02 ppm (30 days) and 0.004 ppm (168 days) (91, 160, 161). The SMAC for methylhydrazine is 0.002 ppm (based on nasal toxicity) at all timepoints from 1 h to 180 days (162). Hydrazine levels in the ISS workspaces were not reported in the NASA Twins Study, which attributed many of the findings to increased exposure to radiation and other stressors (163, 164). The study employed multiple assays to compare the health of a pair of 50-year-old male monozygotic (identical) twin astronauts, one of whom worked in the ISS for 340 days (3.21.15 to 3.21.16) while the other (control) remained earthbound. Significant responses of the twin while working in the ISS included blood cell (CD4, CD8, LD) genomic instability and lengthening of telomers, the chromosomal termini that maintain genomic integrity by preventing inappropriate DNA-damage responses. Although the expression of >90% of in-space modulated genes returned to normal postflight, persistent (at least 6 months) changes were found in (a) the expression of a subset involved with immune function and DNA repair, and (b) the DNA damage from chromosomal inversions, increased number of short telomers, and attenuated cognitive function that was acquired while in space. Telomer maintenance resulting in lengthening (recorded in flight) is frequently activated in tumors of mesenchymal or neuroepithelial origin (165), and telomere shortening (found post-flight) has been associated with long-term exposure to particulate air pollution, certain chemicals including *N*-nitrosamines) decreased telomerase activity and increased cellular aging (166, 167). Ionizing radiation has a bell-shaped dose-response relationship with telomere shortening at low dose (166). Mission-associated mutations of cell-free DNA were found in blood, and increases were noted in biomarkers of inflammation and in serum folate levels, which were correlated with inflight telomere lengthening (163, 164). Tetrahydrofolate, the active form of folate, can damage DNA via metabolic decomposition to formaldehyde (66, 168, 169), the common metabolite of a DNA-damaging hydrazine [60] and of both BMAA and MAM, a radiomimetic substance (17, 19, 170, 171).

Unknown are the cause(s) and significance of the 6-month persistent changes in the twin astronaut who returned to Earth after one year of ISS service. For the following reasons, the possibility of continuous inflight exposure to extremely low levels of UDMH might be considered, along with the astronaut's gene acetylation status. Hydrazine toxicity is concentration-dependent and multiple low doses are cumulative. People with a slow acetylator genotype have less functional *N*-acetyltransferase and therefore do not clear hydrazine from the body as rapidly as fast acetylators; they accumulate higher circulating levels of hydrazine and are probably more susceptible to its short- and long-term toxic effects. Hydrazine reduces liver glutathione, inhibits catalase activity, and increases oxidized glutathione, followed by generation of free radicals (methyl, acetyl, hydroxyl, and hydrogen), increased reactive oxygen species, oxidative stress, and increased lipid peroxidation. Data on the neurological effects of hydrazine(s) are limited (172–175): the acute neuroexcitatory effect (seizures) of ingestion of False Morel mushrooms (see above) result principally from the reaction of hydrazine species with pyridoxal 5-phosphate (activated form of pyridoxine) to form a hydrazone that reduces the inhibitory neurotransmitter GABA by decreasing the enzyme activity of glutamic acid decarboxylase (176); however, hydrazines, such as 1,1-dimethylhydrazine that damage tissue (liver, kidney, CNS?) form a reactive nitrosamide which generates methyl free radicals that produce DNA damage (61, 64) (*O*^6^- and *N*^7^-methylguanine adducts (177) which, if unrepaired, are (like MAM) pro-mutagenic in cycling cells and, presumably, trigger degeneration in terminally dividing and post-mitotic nerve cells (17, 19, 22). Of note, male and female hamsters with year-long intermittent inhalation exposure to hydrazine (0.25, 1.0, and 5.0 ppm) showed “pathologic changes characteristic of degenerative disease, including amyloidosis.” Notably, “Using amyloidosis as a criterion, a no-effect level was not achieved in hamsters (178).” “Exposure-related amyloidosis, hemosiderosis, testicular senile atrophy, and bile duct hyperplasia appeared to effect age-related degeneration (162).” The CNS of these animals is not described and may not have been examined.

## Author Contributions

This paper was developed solely by the author and draws on nearly 40 years of collaborative research on ALS/PDC and related disorders. For most of this time-span, I have had the privilege of working in the field and/or laboratory with key research colleagues, notable among which are Valerie Palmer and Glen Kisby, respectively, with whom I have co-authored many papers relevant to the present topic.

### Conflict of Interest Statement

The author declares that the research was conducted in the absence of any commercial or financial relationships that could be construed as a potential conflict of interest.

## Abbreviations

AD: Alzheimer disease
ALS/PDC: Western Pacific amyotrophic lateral sclerosis and parkinsonism-dementia
L-BMAA: beta-*N*-methylamino-L-alanine
DDR: DNA Damage Response
FUS: Fused in Sarcoma
FTD: Frontotemporal degeneration
MAM: Methylazoxymethanol
MFH: *N*-Methyl-*N*-formylhydrazine
MGMT: *O*^6^-Methylguanine methyltransferase
MMH: Monomethyhydrazine
NDEA: *N*-Nitrosodiethylamine
*O*^6^-mG: *O*^6^-Methylguanine
PSP: Progressive supranuclear palsy
STZ: Streptozotocin
TSP-43: TAR DNA-binding protein 43.

## References

[1] Hirano H [Lessons from Guam ALS/PDC study]. Rinsho Shinkeigaku. (2007) 47:717–21. [In Japanese]. 18210783

[2] Spencer PS Palmer VS Kisby GE. Seeking environmental causes of neurodegenerative disease and envisioning primary prevention. Neurotoxicology. (2016) 56:269–83. 10.1016/j.neuro.2016.03.017 27050202

[3] Kurland L Molgaard CA Guamanian ALS: hereditary or acquired? Adv Neurol. (1982) 36:165–71. 7180682

[4] Kuzuhara S [ALS-parkinsonism-dementia complex of the Kii peninsula of Japan (Muro disease). Historical review, epidemiology and concept]. Rinsho Shinkeigaku. (2007) 47:962–5. [In Japanese]. 18210849

[5] Garruto RM Gajdusek DC Chen K-M. Amyotrophic lateral sclerosis among Chamorro migrants from Guam. Ann Neurol. (1980) 8:612–9. 10.1002/ana.410080612 7212649

[6] Garruto RM Gajdusek DC Chen K-M. Amyotrophic lateral sclerosis and parkinsonism-dementia among Filipino migrants to Guam. Ann Neurol. (1981) 10:341–50. 10.1002/ana.410100405 7316487

[7] Tsunoda K Yamashita T Shimada H Nomura E Takahashi Y Shang J. A migration case of Kii amyotrophic lateral sclerosis/parkinsonism dementia complex with the shortest stay in the endemic area and the longest incubation to develop the disease. J Clin Neurosci. (2017) 46:64–7. 10.1016/j.jocn.2017.08.057 28890043

[8] Shiraki H Yase Y ALS in Japan. In: Vinken PJ Bruyn GW, editors. Handbook of Clinical Neurology, Vol. 22. System Disorders and Atrophy, Part 2. New York, NY: American Elsevier (1975). p. 353.

[9] Morimoto S Hatsuta H Kokubo Y Nakano Y Hasegawa M Yoneda M. Unusual tau pathology of the cerebellum in patients with amyotrophic lateral sclerosis/parkinsonism-dementia complex from the Kii Peninsula, Japan. Brain Pathol. (2018) 28:287–91. 10.1111/bpa.12500 28236345 PMC8028275

[10] Jones M Yang M Mickelsen O. Effects of methylazoxymethanol glucoside and methylazoxymethanol acetate on the cerebellum of the postnatal Swiss albino mouse. Fed Proc. (1972) 31:1508–11. 5056175

[11] Spencer PS. Guam ALS/Parkinsonism-dementia: a long-latency neurotoxic disorder caused by slow toxin(s) in food? Can J Neurol Sci. (1987) 14:347–57. 10.1017/S0317167100037732 3315142

[12] Whiting MG Toxicity of Cycads. Implications for Neurodegenerative Diseases and Cancer. In: Transcript of Four Cycad Conferences. New York, NY: Third World Medical Research Foundation (1988).

[13] Spencer PS Gardner E Palmer VS Kisby GE Environmental neurotoxins linked to a prototypical neurodegenerative disease. In: Aschner M Costa L, editors. Environmental Factors in Neurodevelopment and Neurodegenerative Disorders. New York, NY: Academic Press (2015). p. 212–37. 10.1016/C2013-0-13407-1

[14] Román GC. Neuroepidemiology of amyotrophic lateral sclerosis: clues to aetiology and pathogenesis. J Neurol Neurosurg Psychiatr. (1996) 61:131–7. 10.1136/jnnp.61.2.131 8708678 PMC1073984

[15] Kisby GE Olivas A Park T Churchwell M Doerge D Samson LD. DNA repair modulates the vulnerability of the developing brain to alkylating agents. DNA Repair. (2009) 8:400–12. 10.1016/j.dnarep.2008.12.002 19162564 PMC2692311

[16] Potjewyd G Day PJ Shangula S Margison GP Povey AC. L-β-N-Methylamino-l-alanine (BMAA) nitrosation generates a cytotoxic DNA damaging alkylating agent: an unexplored mechanism for neurodegenerative disease. Neurotoxicology. (2017) 59:105–9. 10.1016/j.neuro.2017.01.007 28163087

[17] Kisby GE Spencer PS. Is neurodegenerative disease a long-latency response to early-life genotoxin exposure? Int J Environ Res Public Health. (2011) 8:3889–921. 10.3390/ijerph8103889 22073019 PMC3210588

[18] Armon C. From Snow to Hill to ALS: an epidemiological odyssey in search of ALS causation. J Neurol Sci. (2018) 391:134–40. 10.1016/j.jns.2018.05.016 29807753

[19] Spencer PS. Formaldehyde, DNA damage, ALS and related neurodegenerative diseases. J Neurol Sci. (2018) 391:141–2. 10.1016/j.jns.2018.05.017 29853250

[20] Chen J Huang XF. The signal pathways in azoxymethane-induced colon cancer and preventive implications. Cancer Biol Ther. (2009) 8:1313–7. 10.4161/cbt.8.14.8983 19502780

[21] Sohn OS Fiala ES Requeijo SP Weisburger JH Gonzalez FJ. Differential effects of CYP2E1 status on the metabolic activation of the colon carcinogens azoxymethane and methylazoxymethanol. Cancer Res. (2001) 61:8435–40. 11731424

[22] Kisby GE Fry RC Lasarev MR Bammler TK Beyer RP Churchwell M. The cycad genotoxin MAM modulates brain cellular pathways involved in neurodegenerative disease and cancer in a DNA damage-linked manner. PLoS ONE. (2011) 6:e20911. 10.1371/journal.pone.0020911 21731631 PMC3121718

[23] Kisby G Palmer V Lasarev M Fry R Iordanov M Magun E. Does the cycad genotoxin MAM implicated in Guam ALS-PDC induce disease-relevant changes in mouse brain that includes olfaction? Commun Integr Biol. (2011) 4:731–4. 10.4161/cib.17603 22446540 PMC3306344

[24] Bennett RA Pegg AE. Alkylation of DNA in rat tissues following administration of streptozotocin. Cancer Res. (1981) 41:2786–90. 6454479

[25] Grieb P. Intracerebroventricular streptozotocin injections as a model of Alzheimer's disease: in search of a relevant mechanism. Mol Neurobiol. (2016) 53:1741–52. 10.1007/s12035-015-9132-3 25744568 PMC4789228

[26] Li D Huang Y Cheng B Su J Zhou WX Zhang YX. Streptozotocin induces mild cognitive impairment at appropriate doses in mice as determined by long-term potentiation and the Morris Water Maze. J Alzheimers Dis. (2016) 54:89–98. 10.3233/JAD-150979 27472873

[27] Ravelli KG Rosário BD Camarini R Hernandes MS Britto LR. Intracerebroventricular streptozotocin as a model of Alzheimer's disease: neurochemical and behavioral characterization in mice. Neurotox Res. (2017) 31:327–33. 10.1007/s12640-016-9684-7 27913964

[28] Zhang Y Ding R Wang S Ren Z Xu L Zhang X. Effect of intraperitoneal or intracerebroventricular injection of streptozotocin on learning and memory in mice. Exp Ther Med. (2018) 16:2375–80. 10.3892/etm.2018.6487 30186480 PMC6122483

[29] Lester-Coll N Rivera EJ Soscia SJ Doiron K Wands JR de la Monte SM. Intracerebral streptozotocin model of type 3 diabetes: relevance to sporadic Alzheimer's disease. J Alzheimers Dis. (2006) 9:13–33. 10.3233/JAD-2006-9102 16627931

[30] Muramatsu K Ikutomo M Tamaki T Shimo S Niwa M. Effect of streptozotocin-induced diabetes on motor representations in the motor cortex and corticospinal tract in rats. Brain Res. (2018) 1680:115–26. 10.1016/j.brainres.2017.12.016 29273401

[31] Tamaki T Muramatsu K Ikutomo M Oshiro N Hayashi H Niwa M. Effects of streptozotocin-induced diabetes on leg muscle contractile properties and motor neuron morphology in rats. Anat Sci Int. (2018) 93:502–13. 10.1007/s12565-018-0444-z 29876845

[32] De la Monte SM Tong M. Mechanisms of nitrosamine-mediated neurodegeneration: potential relevance to sporadic Alzheimer's disease. J Alzheimers Dis. (2009) 17:817–25. 10.3233/JAD-2009-1098 19542621 PMC4550318

[33] Lijinsky W. *N*-Nitroso compounds in the diet. Mutat Res. (1999) 443:129–38. 10.1016/S1383-5742(99)00015-0 10415436

[34] Hotchkiss JH. Preformed *N*-nitroso compounds in foods and beverages. Cancer Surv. (1989) 8:295–321. 2696582

[35] Scanlan RA. Formation and occurrence of nitrosamines in food. Cancer Res. (1983) 43:2435s−40s. 6831466

[36] Wang H O'Reilly ÉJ Weisskopf MG Logroscino G McCullough ML Thun MJ Smoking and risk of amyotrophic lateral sclerosis: a pooled analysis of five prospective cohorts. Arch Neurol. (2011) 68:207–13. 10.1001/archneurol.2010.367 21320987 PMC3319086

[37] Weisskopf MG Gallo V O'Reilly EJ Vineis P Ascherio A. Smoking may be considered an established risk factor for sporadic ALS. Neurol. (2010) 74:1927–8. 10.1212/WNL.0b013e3181e038e9 20549870

[38] Korner S Kammeyer J Zapf A Kuzma-Kozakiewicz M Piotrkiewicz M Kuraskiewcz B. Influence of environment and lifestyle on incidence and progress of amyotrophic lateral sclerosis in a German ALS population. Aging Dis. (2019) 10:205–16. 10.14336/AD.2018.0327 31011473 PMC6457054

[39] Cataldo JK Prochaska JJ Glantz SA. Cigarette smoking is a risk factor for Alzheimer's disease: an analysis controlling for tobacco industry affiliation. J Alzheimers Dis. (2010) 19:465–80. 10.3233/JAD-2010-1240 20110594 PMC2906761

[40] Durazzo TC Meyerhoff DJ Yoder KK. Cigarette smoking is associated with cortical thinning in anterior frontal regions, insula and regions showing atrophy in early Alzheimer's Disease. Drug Alcohol Depend. (2018) 192:277–84. 10.1016/j.drugalcdep.2018.08.009 30300802 PMC6602071

[41] Ma B Stepanov I Hecht SS. Recent studies on DNA adducts resulting from human exposure to tobacco smoke. Toxics. (2019) 7:E16. 10.3390/toxics7010016 30893918 PMC6468371

[42] Belli S Vanacore N. Proportionate mortality of Italian soccer players: is amyotrophic lateral sclerosis an occupational disease? Eur J Epidemiol. (2005) 20:237–42. 10.1007/s10654-004-6879-7 15921041

[43] Chiò A Benzi G Dossena M Mutani R Mora G. Severely increased risk of amyotrophic lateral sclerosis among Italian professional football players. Brain. (2005) 128:472–6. 10.1093/brain/awh373 15634730

[44] Taioli E. All causes of mortality in male professional soccer players. Eur J Publ Health. (2007) 17:600–4. 10.1093/eurpub/ckm035 17434875

[45] Chio A Calvo A Dossena M Ghiglione P Mutani R Mora G. ALS in Italian professional soccer players: the risk is still present and could be soccer-specific. Amyotroph Lateral Scler. (2009) 10:205–9. 10.1080/17482960902721634 19267274

[46] Vanacore N Barbariol P Caffari B Lacorte E Bacigalupo I Spila Alegiani S. Amyotrophic lateral sclerosis and soccer: an internet survey of 29 Italian players. Ann Ist Super Sanita. (2018) 54:364–9. 10.4415/ANN_18_04_14 30575574

[47] Abel EL. Football increases the risk for Lou Gehrig's disease, amyotrophic lateral sclerosis. Percept Mot Skills. (2007) 104:1251–4. 10.2466/pms.104.4.1251-1254 17879657

[48] Wicks P Ganesalingham J Collin C Prevett M Leigh NP Al-Chalabi A. Three soccer playing friends with simultaneous amyotrophic lateral sclerosis. Amyotroph Lateral Scler. (2007) 8:177–9. 10.1080/17482960701195220 17538780

[49] Landry G Murphy T Athletic Field Management. Available online at: https://www.researchgate.net/publication/267975191_ATHLETIC_FIELD_MANAGEMENT (accessed April, 2019).

[50] Wolfe NL Zepp RG Gordon JA Fincher RC. N-nitrosamine formation from atrazine. Bull Environ Contam Toxicol. (1976) 15:242–7. 10.1007/BF01812647 5165

[51] Wei H-R Rhoades MG Shea PJ Formation, adsorption, and stability of *N*-nitrosoatrazine in water and soil. In: Benvenuto MA Roberts-Kirchhoff ES Murray MN Garshott DM, editors. It's All in the Water: Studies of Materials and Conditions in Fresh and Salt Water Bodies. Washington, DC: ACS Symposium Series; American Chemical Society (2001). p. 3–19. 10.1021/bk-2011-1086.ch001

[52] Spiegelharder B Preussmann R Nitrosamines and rubber. IARC Sci Publ. (1982) 41:231–43. 7141533

[53] van Bruggen M van Putten EM Janssen PCJM Nitrosamines Released From Rubber Crumb. RIVM Report 609300002/2007. Available online at: https://core.ac.uk/display/58773540 (accessed April, 2019).

[54] U.S. Environmental Protection Agency. Federal Research on Recycled Tire Crumb Used On Playing Fields. Available online at: https://www.epa.gov/chemical-research/federal-research-recycled-tire-crumb-used-playing-fields (accessed April, 2019).

[55] Karlson-Stiber C Persson H. Cytotoxic fungi—an overview. Toxicon. (2003) 42:339–49. 10.1016/S0041-0101(03)00238-1 14505933

[56] Cornish HH. The role of vitamin B6 in the toxicity of hydrazines. Ann NY Acad Sci. (1969) 166:136–45. 10.1111/j.1749-6632.1969.tb54264.x 5262010

[57] Gamberini M Leite LC. Carbon-centered free radical formation during the metabolism of hydrazine derivatives by neutrophils. Biochem Pharmacol. (1993) 45:1913–9. 8388211 10.1016/0006-2952(93)90451-2

[58] Hawks A Hicks RM Holsman JW Magee PN. Morphological and biochemical effects of 1,2-dimethylhydrazine and 1-methylhydrazine in rats and mice. Br J Cancer. (1974) 30:429–39. 10.1038/bjc.1974.217 4469195 PMC2009305

[59] Hawks A Magee PN. The alkylation of nucleic acids of rat and mouse *in vivo* by the carcinogen 1,2-dimethylhydrazine. Br J Cancer. (1974) 30:440–7. 10.1038/bjc.1974.218 4469196 PMC2009313

[60] Braun R Greeff U Netter KJ. Liver injury by the false morel poison gyromitrin. Toxicology. (1979) 12:155–63. 10.1016/0300-483X(79)90042-8 473232

[61] Braun R Greeff U Netter KJ. Indications for nitrosamide formation from the mushroom poison gyromitrin by rat liver microsomes. Xenobiotica. (1980) 10:557–64. 10.3109/00498258009033790 7445522

[62] Bieganski T Braun R Kusche J. *N*-Methyl-*N*-formylhydrazine: a toxic and mutagenic inhibitor of the intestinal diamine oxidase. Agents Actions. (1984) 14:351–5. 10.1007/BF01973825 6428190

[63] Sedgwick B. Oxidation of methylhydrazines to mutagenic methylating derivatives and inducers of the adaptive response of *Escherichia coli* to alkylation damage. Cancer Res. (1992) 52:3693–7. 1617641

[64] Bergman K Hellenäs KE. Methylation of rat and mouse DNA by the mushroom poison gyromitrin and its metabolite monomethylhydrazine. Cancer Lett. (1992) 61:165–70. 10.1016/0304-3835(92)90175-U 1730140

[65] Toth B. Hepatocarcinogenesis by hydrazine mycotoxins of edible mushrooms. J Toxicol Environ Health. (1979) 5:193–202. 10.1080/15287397909529744 572876

[66] Bosan WS Lambert CE Shank RC. The role of formaldehyde in hydrazine-induced methylation of liver DNA guanine. Carcinogenesis. (1986) 7:413–8. 10.1093/carcin/7.3.413 3948326

[67] Lagrange E Bonneterre V Talbot K Couratier P Salachas F Bernard E Camu W A High-Incidence Cluster of ALS in the French Alps: Common Environment and Multiple Exposures. RISE2017, Strasbourg, France. Available online at: https://core.ac.uk/display/143961926. See also Buguet A, Spencer P, Reis J. Second International Meeting on Environmental Health, Nov. 29-December 1, 2017. World Neurology (2018) 33:8 Available online at: https://worldneurologyonline.com/article/second-international-meeting-on-environmental-health/ (accessed April, 2019).

[68] Spencer PS Lagrange E Camu W. ALS and environment: clues from spatial clustering? Rev Neurol. (2019). 10.1016/j.neurol.2019.04.007 [Epub ahead of print]. 31230725

[69] Born D Real vs. False Morel Mushrooms: Can You Tell the Difference? (2016). Available on line at: https://www.wideopenspaces.com/real-and-false-morel-mushrooms-can-you-tell-the-difference/ (accessed April, 2019).

[70] Anon Fact Sheet: False Morels vs. True Morels. Michigan Department of Community Health. May 23, 2011.

[71] Jokelainen M. The epidemiology of amyotrophic lateral sclerosis in Finland. A study based on the death certificates of 421 patients. J Neurol Sci. (1976) 29:55–63. 10.1016/0022-510X(76)90080-0 950575

[72] Sabel CE Boyle PJ Löytönen M Gatrell AC Jokelainen M Flowerdew R. Spatial clustering of amyotrophic lateral sclerosis in Finland at place of birth and place of death. Am J Epidemiol. (2003) 157:898–905. 10.1093/aje/kwg090 12746242

[73] Jokelainen M Palo J ALS in Finland: birthplaces of patients, parents and grandparents. Acta Neurol Scand. (1980) 62:176–9. 10.1111/j.1600-0404.1980.tb03019.x 7211167

[74] Jokelainen M Wikström J Palo J. Effect of birthplace on the development of amyotrophic lateral sclerosis and multiple sclerosis: a study among Finnish war evacuees. Acta Neurol Scand. (1979) 60:283–8. 10.1111/j.1600-0404.1979.tb02983.x 317413

[75] Rautavaara T Suomen Sienisato [Studies on the Mushroom Crop in Finland and its Utilization]. Porvoo: Werner Söderström Osakeyhtiö (1947). p. 534.

[76] Eiser AR. Why does Finland have the highest dementia mortality rate? Environmental factors may be generalizable. Brain Res. (2017) 1671:14–7. 10.1016/j.brainres.2017.06.032 28687259

[77] Feldman E In: Shamus KJ. *New Study: Michigan's Manufacturing Legacy May Be Affecting our Health, Environment*. Detroit Free Press (2019). Available online at: https://www.freep.com/story/news/health/2019/03/20/study-michigans-manufacturing-legacy-may-affect-health-environment/3134591002/ (accessed April, 2019).

[78] Anon On Cooking False Morels–Gyromitra. Forager Chef. Available online at: https://foragerchef.com/on-cooking-false-morels-gyromitra/ (accessed April, 2019).

[79] Hatten BW McKeown NJ Hendrickson RG Horowitz BZ The spatial epidemiology of toxic mushroom ingestions in the United States: 2001–2011. 2012 Annual Meeting of the North American Congress of Clinical Toxicology (NACCT) October 1–6, 2012 Las Vegas, NV, USA. Clin Toxicol. (2012) 50:574–5. 10.3109/15563650.2012.700015

[80] Yu Y Su FC Callaghan BC Goutman SA Batterman SA Feldman EL. Environmental risk factors and amyotrophic lateral sclerosis (ALS): a case-control study of ALS in Michigan. PLoS ONE. (2014) 9:e101186. 10.1371/journal.pone.0101186 24979055 PMC4076303

[81] Sienko DG Davis JP Taylor JA Brooks BR. Amyotrophic lateral sclerosis. A case-control study following detection of a cluster in a small Wisconsin community. Arch Neurol. (1990) 47:38–41. 10.1001/archneur.1990.00530010046017 2294892

[82] Garcia HD James JT Chapter 9: B5 Hydrazine. In: Spacecraft Maximum Allowable Concentrations for Selected Airborne Contaminants. Vol. 2. Washington, DC: National Academies Press (1996). p. 213–33. Available online at: https://www.nap.edu/read/5170/chapter/9 (accessed April 2019).

[83] Nufer BM A summary of NASA and USAF hypergolic propellant related spills and fires. NASA/TP-2009–214769 (2009). Available online at: https://archive.org/stream/NASA_NTRS_Archive_20090029348/NASA_NTRS_Archive_20090029348_djvu.txt (accessed April, 2019).

[84] Anon Titan II Missile Propellants and Their Break Down Chemicals. Titan II Missile Veterans Health and Wellness. Available online at: https://sites.google.com/site/titan2vetshealthandwellness/rocket-propellants-info/propellants-and-their-breakdown-chemicals (accessed April, 2019).

[85] Anon Pioneer 10. National Air and Space Administration (NASA) Wiki. Fandom, undated. Available online at: https://nasa.fandom.com/wiki/Pioneer_10 (accessed April, 2019).

[86] Uney PE Fester DA Material Compatibility With Space Storable Propellants. Design guidebook. MCR-72–26. Martin Marietta Corp, Denver, Colorado (1972).

[87] Goldstein E The greening of satellite propulsion. Aerosp Am. (2002) 50:25–8.

[88] Young LA Derby MR Aiken EW Use of vertical lift planetary aerial vehicles for the exploration of Mars. In: Concepts and Approaches for Mars Exploration. Available online at: https://www.lpi.usra.edu/meetings/marsconcepts2012/; see also: https://www.researchgate.net/publication/24328376_Mars_Exploration_Using_Vertical-Lift_Planetary_Aerial_Vehicles (accessed April, 2019).

[89] Yost BD Perez AD Smallsat Propulsion Survey. In: IPPW Smallsat Workshop. NASA Space Technology Mission Directorate and NASA Science Mission Directorate. University of Boulder, Colorado (2018). Available online at: https://www.colorado.edu/event/ippw2018/shortcourse-smallsats (accessed July, 2019).

[90] NASA National Aeronautics and Space Administration. Publication List 1979–2018. Available online at: https://amesteam.arc.nasa.gov/Publications/nai_arc_publications.html#1979 (accessed April, 2019).

[91] Noack M NASA Ames Workers Worry Over Superfund Site's Toxins. Mountain View Voice (2016). Available online at: https://mv-voice.com/news/2016/10/28/nasa-ames-workers-worry-over-superfund-sites-toxins (accessed April, 2019).

[92] Laws R Evaluation of a Potential Cluster of Illnesses Among Employees at NASA Ames Research Center. Moffett Field, CA: California Department of Health (2018). p. 13. See also: Hoge P. Barry M Leiner-Senior Computer Scientist at NASA. San Francisco, CA (2003). Available online at: https://www.sfgate.com/bayarea/article/Barry-M-Leiner-seniorcomputer-scientist-at-2621021.php (accessed April, 2019).

[93] Christensen WD An Evaluation of Occupational Exposure to Hydrazine (h-70) During Routine Maintenance Tasks Associated With the F-16 Emergency Power Unit. USAF Occupational and Environmental Health Laboratory Technical Report 78–23, Texas (1978).

[94] Suggs HJ Luskus LJ Kilian HJ Mokry JW Exhaust Gas Composition of the F-16 Emergency Power Unit. SAM 2R. USAF School of Aerospace Medicine, Texas (1979). p. 14.

[95] Stewart C Hydrazine Response Team Hones Skills. 114^th^ Fighter Wing (2014). Available online at: https://www.114fw.ang.af.mil/News/114FW-News/Article/863976/hydrazine-response-team-hones-skills/ (accessed April, 2019).

[96] Associated Press F-16 Chemical Leak Sends 6 Airmen to Hospital. (2016). Available online at: https://www.airforcetimes.com/pay-benefits/military-benefits/2016/08/26/f-16-chemical-leak-sends-6-airmen-to-hospital/ (accessed April, 2019).

[97] Horner RD Kamins KG Feussner JR Grambow SC Hoff-Lindquist J Harati Y. Occurrence of amyotrophic lateral sclerosis among Gulf War veterans. Neurology. (2003) 61:742–9. 10.1212/01.WNL.0000069922.32557.CA 14504315

[98] Spencer M Gulf War Pilot Dies. Hartford Courant (2005). Available online at: https://www.courant.com/news/connecticut/hc-xpm-2005-07-02-0507020570-story.html (accessed April, 2019).

[99] Defeo T Flashback Friday: The Story of the Leathal Fuel Called Hydrazine. DragXine (2010). Available online at: https://www.dragzine.com/news/flashback-friday-the-story-of-the-leathal-fuel-called-hydrazine/ (accessed April, 2019).

[100] Phil C Nitromethane + hydrazine = Madness. Hokey Message Board. (2005). Available online at: https://www.jalopyjournal.com/forum/threads/nitromethane-hydrazine-madness.71606/ (accessed April, 2019).

[101] Anon Indiana Broadcast Pioneers. Sid Collins. Available online at: https://indianabroadcastpioneers.org/sid-collins/ and https://en.wikipedia.org/wiki/Sid_Collins (accessed April, 2019).

[102] Romines S ALS Funding Once Again a Priority for Student. Herald Tribune, Batesville, Indiana (2018). Available online at: https://www.batesvilleheraldtribune.com/news/local_news/als-funding-once-again-a-priority-for-student/article_58bfc062-a261-5153-88b6-849351501937.html

[103] DW Frank Wall: Canadian Street Rodding Hall of Fame Inductee (2010). Available online at: https://welderseries.com/frank-wall-canadian-street-rodding-hall-of-fame-inductee/ (accessed April, 2019).

[104] Anon Permatex/Follow a Dream Team Tuner Tom Howell to Retire. ARP Racing News (2016). Available online at: https://news.arpracingnews.com/category/follow-a-dream-racing/page/2/ (accessed April, 2019).

[105] Anon Longtime Silver Crown Competitor Bateman Passes Away. USAC Racing January 16 (2017). Available online at: http://www.usacracing.com/news/silver-crown/item/5995-longtime-silver-crown-competitor-bateman-passes-away (accessed April, 2019).

[106] Anon RC Sherman Passes. Competition Plus, Drag Racing's Internet Magazine (2017). Available online at: http://www.competitionplus.com/drag-racing/news/4876-rc-sherman-passes (accessed April, 2019).

[107] Lannuzel A Mecharles S Tressières B Demoly A Alhendi R Hédreville-Tablon MA. Clinical varieties and epidemiological aspects of amyotrophic lateral sclerosis in the Caribbean island of Guadeloupe: a new focus of ALS associated with Parkinsonism. Amyotroph Lateral Scler Frontotemporal Degener. (2015) 16:216–23. 10.3109/21678421.2014.992026 25646866

[108] Caparros-Lefebvre D Sergeant N Lees A Camuzat A Daniel S Lannuzel A. Guadeloupean parkinsonism: a cluster of progressive supranuclear palsy-like tauopathy. Brain. (2002) 125:801–11. 10.1093/brain/awf086 11912113

[109] Lannuzel A Höglinger GU Verhaeghe S Gire L Belson S Escobar-Khondiker M. Atypical parkinsonism in Guadeloupe: a common risk factor for two closely related phenotypes? Brain. (2007) 130:816–27. 10.1093/brain/awl347 17303592

[110] Yamazaki M Makifuchi T Chen KM Mori O Katayama Y Takahashi H. Progressive supranuclear palsy on Guam. Acta Neuropathol. (2001) 102:510–4. 11699567 10.1007/s004010100408

[111] Steele JC Caparros-Lefebvre D Lees AJ Sacks OW. Progressive supranuclear palsy and its relation to pacific foci of the parkinsonism-dementia complex and Guadeloupean parkinsonism. Parkinsonism Relat Disord. (2002) 9:39–54. 10.1016/S1353-8020(02)00043-3 12217621

[112] Caparros-Lefebvre D Steele J Kotake Y Ohta S. Geographic isolates of atypical Parkinsonism and tauopathy in the tropics: possible synergy of neurotoxins. Mov Disord. (2006) 21:1769–71. 10.1002/mds.21024 16874753

[113] Lannuzel A Ruberg M Michel PP. Atypical parkinsonism in the Caribbean island of Guadeloupe: etiological role of the mitochondrial complex I inhibitor annonacin. Mov Disord. (2008) 23:2122–8. 10.1002/mds.22300 18816693

[114] Rottscholl R Haegele M Jainsch B Xu H Respondek G Höllerhage M. Chronic consumption of *Annona muricata* juice triggers and aggravates cerebral tau phosphorylation in wild-type and MAPT transgenic mice. J Neurochem. (2016) 139:624–39. 10.1111/jnc.13835 27569447

[115] Klaus Kubitzki K Rohwer JG Bittrich V Flowering Plants: Dicotyledons: Magnoliid, Hamamelid and Caryophyllid Families. Berlin: Springer-Verlag (1993).

[116] Druckrey H Preussman R lvancovic S Schmähl D. [Organotropic carcinogenic effects of 65 various *N*-nitroso- compounds on BD rats]. Z Krebsforsch. (1967) 69:103–201. 10.1007/BF00524152 [In German]. 4230610

[117] Caparros-Lefebvre D Golbe LI Deramecourt V Maurage CA Huin V Buée-Scherrer V. A geographical cluster of progressive supranuclear palsy in northern France. Neurology. (2015) 85:1293–300. 10.1212/WNL.0000000000001997 26354981 PMC4617163

[118] Lee HL Chang LW Wu JP Ueng YF Tsai MH Hsieh DP. Enhancements of 4-(methylnitrosamino)-1-(3-pyridyl)-1-butanone (NNK) metabolism and carcinogenic risk via NNK/arsenic interaction. Toxicol Appl Pharmacol. (2008) 227:108–14. 10.1016/j.taap.2007.09.024 17976671

[119] Kant R Textile dyeing industry an environmental hazard. Natural Sci. (2012) 4:22–6. 10.4236/ns.2012.41004

[120] Gregory P Toxicology of textile dyes. In: Christie RM editor. Environmental Aspects of Textile Dyeing. Boca Raton, FL: CRC Press (2007). p. 44–73.

[121] Carhonell J Sanahuja V Siegrist H Hydrazinium Dyes, with Maleic Acid or Its Salts and Dyeing Therewith. United States Patent 3,357,782. Basel, Switzerland N0 Drawing. Filed (1964). Available online at: https://patents.google.com/patent/US3357782A/en (accessed April, 2019).

[122] Anon *Hydrazine*. PubChem. US National Library of Medicine. National Center for Biotechnology Information. Available online at: https://pubchem.ncbi.nlm.nih.gov/compound/hydrazine (accessed April, 2019).

[123] Vithanage M Dabrowska BB Mukherjee AB Sandhi A Bhattacharya P Arsenic uptake by plants and possible phytoremediation applications: a brief overview. Environ Chem Lett. (2011) 10:1–8. 10.1007/s10311-011-0349-8

[124] Ojuederie OB Babalola OO. Microbial and plant-assisted bioremediation of heavy metal polluted environments: a review. Int J Environ Res Public Health. (2017) 14:E1504. 10.3390/ijerph14121504 29207531 PMC5750922

[125] Albert Q Baraud F Leleyter L Lemoine M Heutte N Rioult JP Use of soil fungi in the biosorption of three trace metals (Cd, Cu, Pb): promising candidates for treatment technology? Environ Technol. (2019) 29:1–32. 10.1080/09593330.2019.1602170 30924724

[126] Govarthanan M Mythili R Kamala-Kannan S Selvankumar T Srinivasan P Kim H. *In-vitro* bio-mineralization of arsenic and lead from aqueous solution and soil by wood rot fungus, Trichoderma sp. Ecotoxicol Environ Saf. (2019) 174:699–705. 10.1016/j.ecoenv.2019.03.034 30878010

[127] Lahiri VL Khanna P Singh K Elhence BR Wahal PK Nitrosamine in leather dust extracts. Br J Ind Med. (1988) 4:647–8. 10.1136/oem.45.9.647 PMC1009671 3179242

[128] Abarbanel JM Herishanu YO Osimani A Frisher S. Motor neuron disease in textile factory workers. Acta Neurol Scand. (1989) 79:347–9. 10.1111/j.1600-0404.1989.tb03796.x 2728859

[129] Buckley J Warlow C Smith P Hilton-Jones D Irvine S Tew JR. Motor neuron disease in England and Wales, 1959–1979. J Neurol Neurosurg Psychiat. (1983) 46:197–205. 10.1136/jnnp.46.3.197 6842228 PMC1027325

[130] Hawkes CH Fox AJ. Motor neurone disease in leather workers. Lancet. (1981) 1:507. 10.1016/S0140-6736(81)91900-0 6110128

[131] Sutedja NA Veldink JH Fischer K Kromhout H Wokke JHJ Huisman MHB Lifetime occupation, education, smoking, and risk of ALS. Neurology. (2007) 9:1508–14. 10.1212/01.wnl.0000277463.87361.8c 17923612

[132] Konopka A Atkin JD. The emerging role of DNA damage in the pathogenesis of the C9orf72 repeat expansion in amyotrophic lateral sclerosis. Int J Mol Sci. (2018) 19:3137. 10.3390/ijms19103137 30322030 PMC6213462

[133] Jackson SP Bartek J. The DNA-damage response in human biology and disease. Nature. (2009) 461:1071–8. 10.1038/nature08467 19847258 PMC2906700

[134] Fielder E von Zglinicki T Jurk D. The DNA damage response in neurons: die by apoptosis or survive in a senescence-like state? J Alzheimers Dis. (2017) 60:S107–31. 10.3233/JAD-161221 28436392

[135] Mitra J Guerrero EN Hegde PM Liachko NF Wang H Vasquez V Motor neuron disease-associated loss of nuclear TDP-43 is linked to DNA double-strand break repair defects. Proc Natl Acad Sci USA. (2019) 116:4696–705. 10.1073/pnas.1818415116 PMC6410842 30770445

[136] Neumann M Kwong LK Lee EB Kremmer E Flatley A Xu A. Phosphorylation of S409/410 of TDP-43 is a consistent feature in all sporadic and familial forms of TDP-43 proteinopathies. Acta Neuropathol. (2009) 117:137–49. 10.1007/s00401-008-0477-9 19125255 PMC2693625

[137] Mackenzie IR Neumann M. FET proteins in frontotemporal dementia and amyotrophic lateral sclerosis. Brain Res. (2012) 1462:40–3. 10.1016/j.brainres.2011.12.010 22261247

[138] Miklossy J Steele JC Yu S McCall S Sandberg G McGeer EG. Enduring involvement of tau, beta-amyloid, alpha-synuclein, ubiquitin and TDP-43 pathology in the amyotrophic lateral sclerosis/parkinsonism-dementia complex of Guam (ALS/PDC). Acta Neuropathol. (2008) 116:625–37. 10.1007/s00401-008-0439-2 18843496

[139] Mimuro M Yoshida M Kuzuhara S Kokubo Y. Amyotrophic lateral sclerosis and parkinsonism-dementia complex of the Hohara focus of the Kii Peninsula: a multiple proteinopathy? Neuropathology. (2018) 38:98–107. 10.1111/neup.12434 29063640

[140] Geser F Winton MJ Kwong LK Xu Y Xie SX Igaz LM. Pathological TDP-43 in parkinsonism-dementia complex and amyotrophic lateral sclerosis of Guam. Acta Neuropathol. (2008) 115:133–45. 10.1007/s00401-007-0257-y 17713769

[141] Hasegawa M Arai T Akiyama H Nonaka T Mori H Hashimoto T. TDP-43 is deposited in the Guam parkinsonism-dementia complex brains. Brain. (2007) 130:1386–94. 10.1093/brain/awm065 17439983

[142] Penndorf D Witte OW Kretz A. DNA plasticity and damage in amyotrophic lateral sclerosis. Neural Regen Res. (2018) 13:173–80. 10.4103/1673-5374.226377 29557356 PMC5879878

[143] Yoshioka K-I Yashioka Y Hsieh P. ATR kinase activation mediated by MutSα and MutLα in response to cytotoxic *O*^6^-methylguanine adducts. Mol Cell. (2006) 22:501–10. 10.1016/j.molcel.2006.04.023 16713580 PMC2423943

[144] Bosan WS Shank RC. Methylation of liver DNA guanine in hamsters given hydrazine. Toxicol Appl Pharmacol. (1983) 70:324–34. 10.1016/0041-008X(83)90108-4 6623473

[145] Albano E Tomasi A Goria-Gatti L Iannone A. Free radical activation of monomethyl and dimethyl hydrazines in isolated hepatocytes and liver microsomes. Free Radic Biol Med. (1989) 6:3–8. 10.1016/0891-5849(89)90152-4 2536341

[146] Gannett PM Garrett C Lawson T Toth B. Chemical oxidation and metabolism of *N*-methyl-*N*-formylhydrazine. Evidence for diazenium and radical intermediates. Food Chem Toxicol. (1991) 29:49–56. 10.1016/0278-6915(91)90062-C 1999305

[147] Hebels DG Briedé JJ Khampang R Kleinjans JC de Kok TM. Radical mechanisms in nitrosamine- and nitrosamide-induced whole-genome gene expression modulations in Caco-2 cells. Toxicol Sci. (2010) 116:194–205. 10.1093/toxsci/kfq121 20403970

[148] Gamberini M Leite LC. Carbon-centered free radical formation during the metabolism of hydrazine derivatives by neutrophils. Biochem Pharmacol. (1993) 45:1913–9. 8388211 10.1016/0006-2952(93)90451-2

[149] Spencer PS Ohta M Palmer VS. Cycad use and motor neurone disease in Kii Peninsula of Japan. Lancet. (1987) 2:1462–3. 10.1016/S0140-6736(87)91159-7 2892020

[150] Christmann M Verbeek B Roos WP Kaina B. O(6)-Methylguanine-DNA methyltransferase (MGMT) in normal tissues and tumors: enzyme activity, promoter methylation and immunohistochemistry. Biochim Biophys Acta. (2011) 1816: 179–90. 10.1016/j.bbcan.2011.06.002 21745538

[151] Iyama T Wilson DM III. DNA repair mechanisms in dividing and non-dividing cells. DNA Repair. (2013) 12:620–36. 10.1016/j.dnarep.2013.04.015 23684800 PMC3720834

[152] Christmann M Kaina B MGMT- A critical DNA repair gene target for chemotherapy resistance. In: Kelley MR Fishel ML, editors. DNA Repair in Cancer Therapy. Molecular Targets and Clinical Applications. 2nd ed. Amsterdam: Elsevier (2016). p. 55–82. 10.1016/B978-0-12-803582-5.00002-4

[153] Goth R Rajewsky MF. Persistence of *O*^6^-ethylguanine in rat-brain DNA: correlation with nervous system-specific carcinogenesis by ethylnitrosourea. Proc Natl Acad Sci USA. (1974) 71:639–43. 10.1073/pnas.71.3.639 4522778 PMC388067

[154] Kleihues P Bucheler J Long-term persistence of *O*^6^-methylguanine in rat brain DNA. Nature. (1977) 26:625–6. 10.1038/269625a0 917114

[155] Kleihues P Cooper HK Buecheler J Kolar GF Diessner H Mechanism of perinatal tumor induction by neuro-oncogenic alkylnitrosoureas and dialkylaryltriazenes. Natl Cancer Inst Monogr. (1979) 51:227–31. 481575

[156] Roos WP Kaina B. DNA damage-induced apoptosis: from specific DNA lesions to the DNA damage response and apoptosis. Cancer Lett. (2012) 332:237–48. 10.1016/j.canlet.2012.01.007 22261329

[157] Liu D Liu C Li J Azadzoi K Yang Y Fei Z. Proteomic analysis reveals differentially regulated protein acetylation in human amyotrophic lateral sclerosis spinal cord. PLoS ONE. (2013) 8:e80779. 10.1371/journal.pone.0080779 24312501 PMC3846615

[158] Chen J Huang W Cheng CH Zhou L Hu YY. Association between aldehyde dehydrogenase-2 polymorphisms and risk of Alzheimer's Disease and Parkinson's Disease: a meta-analysis based on 5,315 individuals. Front Neurol. (2019) 10:290. 10.3389/fneur.2019.00290 30984100 PMC6448532

[159] Derks KWJ Hoeijmakers JHJ Pothof J. The DNA damage response: the omics era and its impact. DNA Repair. (2014) 19:214–20. 10.1016/j.dnarep.2014.03.008 24794401 PMC4088957

[160] National Research Council Chapter 6: Hydrazine. In: Emergency and Continuous Exposure Guidance Levels for Selected Submarine Contaminants. Vol. 1 Washington, DC: National Academies Press (2007). p. 139–66. 10.17226/11170

[161] National Academies of Sciences Engineering and Medicine Refinements to the Methods for Developing Spacecraft Exposure Guidelines. Washington, DC: National Academies Press (2016). p. 64 10.17226/21888 27252994

[162] National Research Council Spacecraft Maximum Allowable Concentrations for Selected Airborne Contaminants: Volume 4. Washington, DC: The National Academies Press (2000). p. 119–36. 10.17226/9786

[163] Garrett-Bakelman FE Darshi M Green SJ Gur RC Lin L Macias BR. The NASA twins study: a multidimensional analysis of a year-long human spaceflight. Science. (2019) 364:eaau8650. 10.1126/science.aau8650 30975860 PMC7580864

[164] NASA NASA Twins Study Investigators to Release Integrated Paper in 2019. Available online at: https://www.nasa.gov/feature/nasa-twins-study-investigators-to-release-integrated-paper-in-2019 (accessed July 11, 2019).

[165] Sobinoff AP Pickett HA. Alternative lengthening of telomeres: DNA repair pathways converge. Trends Genet. (2017) 33:921–32. 10.1016/j.tig.2017.09.003 28969871

[166] Møller P Wils RS Jensen DM Andersen MHG Roursgaard M. Telomere dynamics and cellular senescence: an emerging field in environmental and occupational toxicology. Crit Rev Toxicol. (2018) 48:761–88. 10.1080/10408444.2018.1538201 30570381

[167] Zhang X Lin S Funk WE Hou L. Environmental and occupational exposure to chemicals and telomere length in human studies. Postgrad Med J. (2013) 89:722–8. 10.1136/postgradmedj-2012-101350rep 24243983

[168] Burgos-Barragan G Wit N Meiser J Dingler FA Pietzke M Mulderrig L Mammals divert endogenous genotoxic formaldehyde into one-carbon metabolism. Nature. (2017) 548:9–54. 10.1038/nature23481 PMC5714256 28813411

[169] MRC Laboratory of Molecular Biology Folate and Formaldehyde: From Vitamin to Genotoxin to DNA Building Block. Available online at: https://www2.mrc-lmb.cam.ac.uk/folate-and-formaldehyde-from-vitamin-to-genotoxin-to-dna-building-block/ (accessed July 11, 2019).

[170] Teas HJ Sax HJ Sax K. Cycasin: radiomimetic effect. Science. (1965) 149:541–2. 14325155 10.1126/science.149.3683.541

[171] Spencer P Fry RC Kisby GE. Unraveling 50-year-old clues linking neurodegeneration and cancer to cycad toxins: are microRNAs common mediators? Front Genet. (2012) 3:192. 10.3389/fgene.2012.00192 23060898 PMC3460211

[172] Kulagina NK The toxicological characteristics of hydrazine. Toxicology of new industrial chemical substances. Acad Med Sci. (1962) 4:65–81.

[173] Reid FJ. Hydrazine poisoning. Br Med J. (1965) 2:1246. 5849139 10.1136/bmj.2.5472.1246-a PMC1846609

[174] ATSDR Toxicological Profile for Hydrazines. Atlanta, GA: Agency for Toxic Substances and Disease Registry, U.S. Public Health Service (1997). p. 185 + appendices. 37797136

[175] Hu WX Tan CY Tan SJ Jiang J Progress in the protective medicine against [correction of aganist] rocket propellents (Chinese). Space Med Med Eng. (1999) 12:451–5. 12434814

[176] Horowitz K Horowitz BZ Toxicity, Mushroom, Gyromitra. Treasure Island, FL: Stat Pearls Intern (2019).

[177] Mathison BH Murphy SE Shank RC. Hydralazine and other hydrazine derivatives and the formation of DNA adducts. Toxicol Appl Pharmacol. (1994) 127:91–8. 8048059 10.1006/taap.1994.1143

[178] Vernot EH MacEwen JD Bruner RH Haun CC Kinkead ER Prentice DE. Long-term inhalation toxicity of hydrazine. Fundam Appl Toxicol. (1985) 5 (6 Pt 1):1050–64. 4092868 10.1016/0272-0590(85)90141-1

